# Prognostic Value of Pleural Lavage Cytology in Patients with Lung Cancer Resection: An Updated Meta-Analysis

**DOI:** 10.1371/journal.pone.0157518

**Published:** 2016-07-26

**Authors:** Chun-Mei Wang, Zhou-Gui Ling, Yan-Bin Wu, Shuang-Qi Cai, Zhen-Ming Tang, Cong Wu, Yi-Qiang Chen

**Affiliations:** 1 Department of Respiratory Diseases, the People's Hospital of Shenzhen Guangming New District, Shenzhen, China; 2 Department of Respiratory Diseases, the Fourth Affiliated Hospital of Guangxi Medical University, Liuzhou, Guangxi, China; 3 Institute of Respiratory Diseases, the First Affiliated Hospital of Guangxi Medical University, Nanning, Guangxi, China; Catalan Institute of Oncology, SPAIN

## Abstract

**Objective:**

Pleural lavage cytology (PLC) is considered as a possible tool for assessing prognosis of lung cancer patients. We aimed to comprehensively review the prognosis value of PLC in patients undergoing surgical resection.

**Methods:**

We searched 4 electronic databases for relevant studies comparing positive PLC and negative PLC. The primary outcomes included survival rate and recurrence rate at maximum follow-up.

**Results:**

The meta-analysis included 28 studies, with a total of 20,714 patients. For the overall survival rate of all stages, the results demonstrated that positive pre-resection, post-resection and pooled PLC were associated with unfavorable survival: hazard ratio (HR) 2.89 (95% confidence interval [CI] 2.48–3.37), 2.70 (1.90–3.83), and 2.89 (2.52–3.31), respectively. For the stage I survival rate, the combined results also suggested that positive pre-resection, post-resection and pooled PLC were associated with unfavorable survival: HR 3.29 (95% CI 2.55–4.25), 4.85 (2.31–10.20), and 3.16 (2.53–3.94), respectively. Furthermore, a meta-analysis of 14 studies included 14,279 patients showed that positive pre-resection, post-resection and pooled PLC were associated with an increased risk of overall recurrence: risk ratio (RR) 2.45 (95% CI 1.91–3.15), 2.37 (1.11–5.09), and 2.37 (95% CI 2.00–2.80), respectively. Positive PLC was also associated with a high pleural recurrence (RR 4.77; 95% CI 3.13–7.26) or distant recurrence (RR 2.33; 95% CI 1.65–3.29).

**Conclusions:**

Both positive pre- resection and post-resection PLC are associated with not only higher tumor recurrence but also unfavorable survival outcomes in patients with lung cancer resection. This technique can therefore act as a strong prognostic factor for tumor recurrence and adverse survival rates.

## Introduction

Lung cancer is a major cause of cancer-related deaths in the Western world [1], and its prognosis closely depends on the disease stage at diagnosis. Early detection and surgical resection are regarded as the mainstay to reduce the mortality of lung cancer patients [2]. However, the recurrence rate remains significant even with complete resection for early stage non-small-cell lung cancer (NSCLC) [3, 4]. One reason might be the exfoliation from the tumor in the pleural cavity, which spreads at a microscopic level even in the early stages [2]. Gu et al.[5] have suggested that occult micrometastatic tumor cells have already spread to the lymph nodes and bone marrow at the time of surgery, even though current clinical staging examinations and conventional histopathologic methods do not detected this. Therefore, methods to detect microscopic spread in patients with lung cancer resection are needed. One of those is pleural lavage cytology (PLC), which is a simple method with the potential to detect microscopic spread.

PLC is a technique that obtains cancer cells from saline instilled into and retrieved from the chest cavity during surgery for non-small-cell lung cancer (patients without preoperative pleural effusion) [6]. A positive PLC indicates the spread of microscopic intrapleural cancer resulting from visceral pleural invasion, vascular invasion or lymphatic permeation of the primary lesion [7]. Since it was first reported by Spjut et al.[8] in 1958, many studies have reported that positive PLC is associated with a poorer prognosis than is negative PLC [9–13]. Two previous meta-analyses concluded that positive PLC is a strong prognostic factor for survival in patients with lung cancer, and patients with PLC-positive disease may be appropriate for upstaging by one T category [6,14]. However, neither of these meta-analyses evaluated the relationship between positive PLC and recurrence, and the study by Lim et al.[6] did not differentiate between pre-resection and post-resection PLC. Recently, another meta-analysis [15] published in 2012, showed that patients with positive pre-resection PLC were associated with not only unfavorable survival outcomes but also higher tumor recurrence; however, the data regarding positive PLC after lung resection were not assessed in this meta-analysis. Since that time, many relevant studies in this field have been published. Some of those included large numbers of patients but conveyed controversial results. Therefore, we performed an updated meta-analysis to comprehensively assess the value of positive PLC in predicting the survival and recurrence rates in patients presenting with lung cancer resection.

## Methods

### Search strategy and inclusion criteria

Relevant studies were obtained by searching the PubMed, Embase, Cochrane Library and Chinese Biomedical Literature on disc (CBM) databases through September 15, 2015. The search terms for all four databases included (lung cancer OR pulmonary cancer OR lung neoplasm OR non-small-cell lung cancer) AND “Pleural lavage”, AND (lung resection OR pulmonary resection) in the title, abstract and keywords. The search was expanded using the ‘related articles’ function and by considering reference lists of all studies, including review articles. The search results were limited to human subjects. Additional articles were also identified by manually searching the references cited in the articles. This process was performed repeatedly until no additional articles could be found. Initially there were no language restrictions, but the full-text review and final analysis were limited to articles published in English and Chinese. The review was limited to the published studies. If evidence indicated that some patients participated in multiple studies (e.g., two or more articles with the same authors, institutions, or period of study), only the most recent article and the best-quality study were selected. Two reviewers (CMW and ZGL) independently judged study eligibility while screening the citations. Disagreements were resolved by a consensus determination by the reviewers.

To be included in our meta-analysis, studies had to meet the following criteria: studies had to compare outcomes related to positive and negative PLC in patients with lung cancer resection and to report either recurrence data or results that would allow the calculation of survival data. Conference abstracts were excluded because of the limited data presented in them. Review articles and studies in which it was not possible to extract the primary outcome of interest were also excluded.

### Data extraction and outcome measurements

Two investigators (CMW and ZGL) independently extracted the following data: first author, year of publication, location, number of patients, total number of positive versus negative PLCs, maximum follow-up, outcomes, and European Lung Cancer Working Party (ELCWP) score. The extracted data were gathered in a standardized Access 2010 (Microsoft Corp) file and checked by another author (YBW). If conflicting results were reported, the data were checked by two authors (CW and YQC) until the disagreement was resolved. If any information was missing from a study, we contacted the authors to obtain the missing data. Some data were extracted from two previous meta-analyses [14,15].

PLC was performed immediately after thoracotomy and before closure of the thoracic cavity in the patients with no pleural effusions or apparent dissemination, then positive and negative pre- or post-resection PLC was compared. To explore the survival rate and recurrence rate of positive PLC, we only extracted data from the maximum follow-up. The outcomes of interest included overall survival rate of all stages, survival rate of stage I, overall recurrence of positive and negative PLC. Survival rates on the graphical representation of survival curves were determined by Engauge Digitizer version 4.1, three independent researchers (CMW, ZGL and ZMT) read the curves to reduce the imprecision in the reading variations.

### Quality assessment

A methodological quality assessment of the included studies was performed by two investigators (CMW and ZGL), who were blinded to the study identities, using the European Lung Cancer Working Party (ELCWP) scale [16]. The description of methods was obtained from one article [14].

### Statistical analysis

The impact of PLC on survival outcome was measured by hazard ratio (HR), which resulted from the survival rate at maximum follow-up. An HR >1 implied a worse survival outcome for the group with positive PLC. The recurrence rate was expressed as a risk ratio (RR) with 95% confidence interval (CI). An RR >1 implied a higher probability of recurrence for the positive PLC group. Heterogeneity of the included studies was evaluated using an I^2^ statistic, and sensitivity analysis was performed to further explore heterogeneity. If there was homogeneity of the individuals, a fixed-effect model was used. When the P-value of the heterogeneity test was <0.1, a random-effect model was used.

To assess the publication bias, we investigated the influence of a single study on the summary effect by omitting the outliers in each turn. The HR was calculated as described previously [17,18], and Log HRs, SE and their variations were then calculated by Review Manager Version 5.3. (The Cochrane Collaboration, Oxford, UK). Analyses were tested using Review Manager version 5.3 and SPSS version 16.0 meta-analysis software. A P-value <0.05 was judged as statistically significant.

## Results

### Study identification and selection

**Fig 1:**
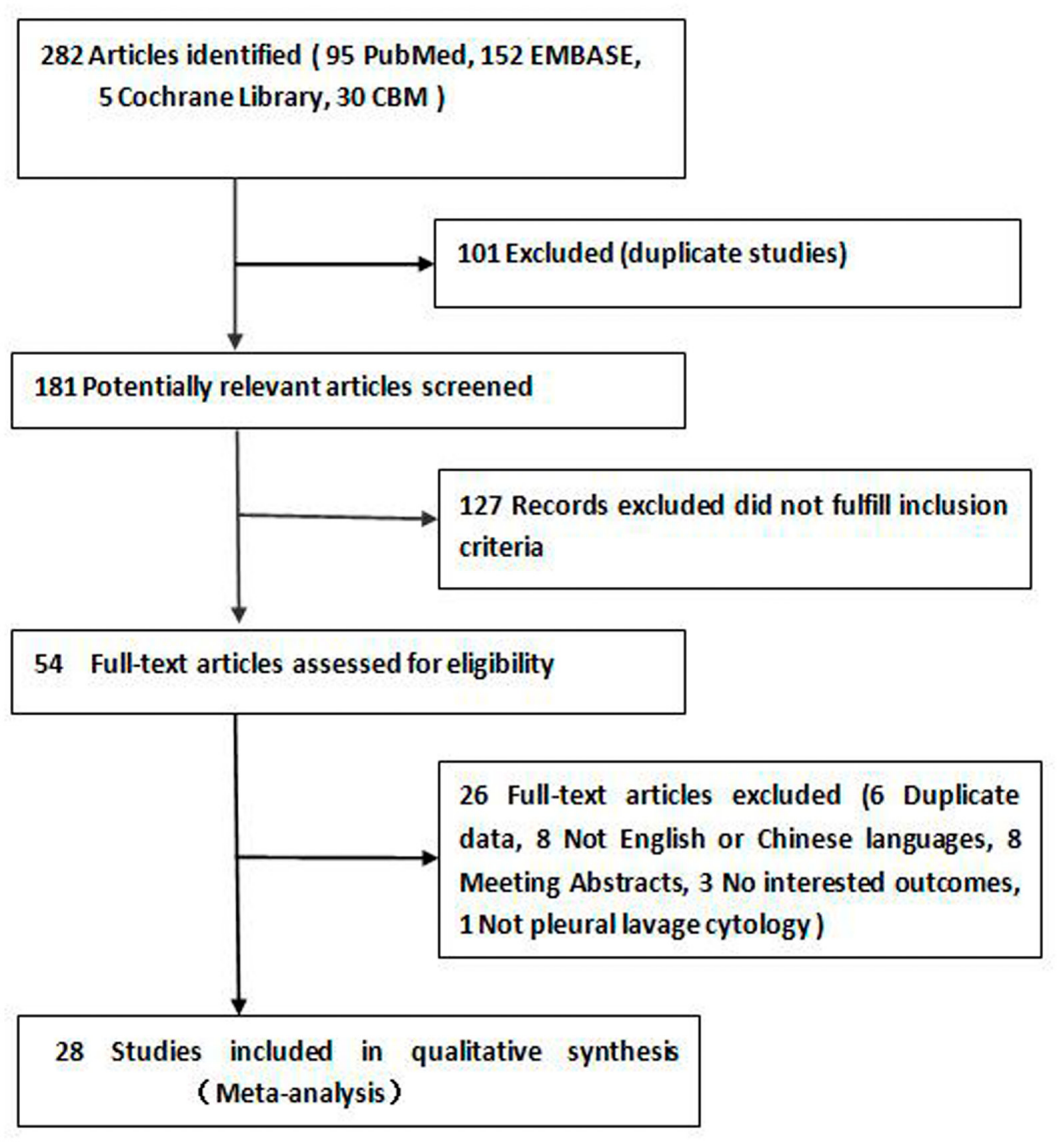
Selection process for the meta-analysis.

After an initial independent search, we identified 282 potentially relevant publications and excluded 101 duplicates. After reviewing the titles and abstracts, we excluded 127 publications because they did not meet the inclusion criteria. Among the remaining 54 full-text articles, 6 were excluded because the same authors published several reports on the same patients, and only the best-quality study was considered [2,19–23]; 3 were excluded because no outcomes of interest were reported [24–26]; 8 were excluded because they were meeting abstracts[27–34]; and 8 were excluded because they were non-English or non-Chinese publications [35–42]. One article was excluded because lavage cytology was not performed in the pleural cavity [43]. Finally, 28 articles were included in the present analysis [7,9,10,12,13,44–66]. The selection process is shown in Fig 1.

### Study characteristics

The meta-analysis included 28 studies involving 20,714 patients in which PLC was performed, and the baseline characteristics of these 28 trials are shown in Table 1. These studies were published between 1990 and 2015. The sample size of the included trials ranged from 84 to 4,171 individuals. Among these studies, 6 were conducted in Europe [10,45,47,49,52,65]; 20 in Japan [7,9,12,13,44,46,50–51,53–57,59–64,66]; and one each in the USA and China [48,58]. Among the 28 studies included here, 22 reported the survival rate of positive PLC versus negative PLC before resection, 5 reported the survival rate after resection, and 2 had PLC data from both pre- and post-resection. There were 14 studies that reported the stage I survival rate of positive PLC versus negative PLC (Table 1), and 14 reported the recurrence rate of positive PLC versus negative PLC (Table 2).

**Table 1:** Survival outcomes of PLC in the meta-analysis.

Author/Year	Location	Patients (N)	Pos/Neg PLC	Maximum follow-up (year)	Overall survival rate (%)		HR	Stage I survival rate (%)		HR	ELCWP score
					Pos/Neg	p-Value		Pos/Neg	p-Value		
**Pre-resection PLC**											
Kondo/1993	Japan	467	42/425	3	22.9/68.7	<0.0001	7.39	NA			70.3
Buhr /1997	Germany	342	132/210	4	24.0/52.0	0.007	3.43	35.0/69.0	0.037	4.13	81.3
Higashiyama/1997	Japan	303	41/262	5	NA			NA			72.8
Hillerdal/1998	Sweden	138	17/121	3	41.2/60.2	>0.05	2.16	NA			56.5
Dresler/1999	USA	124	17/107	2	31.0/63.0	0.088	3.79	48.0/82.0	0.009	4.94	72.8
Lim/2004	UK	292	13/279	3	28.2/ 64.5	0.002	4.63	NA			78.0
Tomita/2005	Japan	150	16/134	5	56.8 / 75.3	0.15	2.32	NA			79.8
Vicidomini/2005	Italy	84	19/65	3	30.0/ 65.0	0.025	4.33	NA			77.8
Nakagawa/2007	Japan	1004	27/977	5	37.0 /69.4	0.007	3.86	21.8/ 86.5	0.001	22.98	84.5
Tomita/2008	Japan	122	9/113	5	33.3/ 83.2	0.001	9.92	NA			79.8
Higashiyama/2009	Japan	679	89/590	5	43.0/66.0	<0.0001	2.57	57.0/ 80.0	<0.0001	3.02	72.8
Kawachi/2009	Japan	568	41/527	5	34.4 / 64.0	0.0001	3.39	NA			81.3
Nakamura/2009	Japan	284	13/271	5	12.3/ 66.0	<0.0001	13.84	NA			76.3
Shintani/2009	Japan	1249	67/1182	5	44.1 / 58.3	0.039	1.77	NA			81.3
Wang/2009	China	172	47/125	5	26.1/ 49.2	<0.001	2.74	NA			67.5
Taniguchi/2009	Japan	281	14/267	5	45.0/ 72.0	0.047	3.14	67.0/ 82.0	0.004	2.24	77.5
Aokage/2010	Japan	2135	65/2070	5	CI:1.71–3.20		2.34	NA			80.0
Hanagiri/2011	Japan	322	13/309	5	54.7/79.0	0.098	3.12	NA			72.5
Kaneda/2012	Japan	3231	148/3083	5	48.6 73.2	<0.001	2.89	56.8/77.0	0.01	2.55	80.0
Baba/2013	Japan	386	17/369	5	38.0/84.0	<0.01	8.57	NA			82.5
Yanagawa/2014	Japan	428	19/409	5	NA			46.6/76.5	<0.004	3.73	86.3
Mazza/2014	Italy	414	15/399	5	35.9/57.8	0.004	2.45	42.9/69.4	0.001	3.02	78.8
Hokka/2015	Japan	1317	46/1271	5	28.0/61.0	<0.0001	4.02	39.5 /77.3	<0.0001	5.22	75.8
Nakao/2015	Japan	1572	56/1516	5	50.6/78.0	<0.001	3.46	50.6 /88.9	<0.001	7.82	72.5
Overall		16,064	983/15081								
**Post-resection PLC**											
Dresler/1999	USA	121	14/107	2	43.0/58.0	0.04	1.67	67.0/80.0	0.97	1.97	72.8
Higashiyama/1997	Japan	306	44/262	5	NA			NA			72.8
Kotoulas/2001	Greece	85	8/77	2	52.0/90.0	0.0081	8.31	NA			66.5
Maruyama/2004	Japan	143	49/94	5	55.1/ 5.1	0.02	2.46	75.0/ 89.9	0.02	2.97	68.3
Taniguchi/2009	Japan	293	26/267	5	32.0/76.0	<0.0001	6.73	33.0/86.0	<0.0001	12.47	77.5
Aokage/2010	Japan	2140	70/2070	5	22.0/ 9.0	<0.001	7.89	NA			80.0
Overall		3088	211/2877								
**Combination of pre- and post-resection PLC**											
Okumura/1991	Japan	158	23/135	3	38.8/69.2	<0.05	3.54	NA			64.5
Kameyama/2014	Japan	4171	217/3954	5	44.5/72.8	<0.0001	3.34	61.5/77.1	0.0062	2.11	66.3
Overall		4,329	240/4089								

HR = [p_0_/(1-p_0_)]/[p_1_/(1-p_1_)], p_0_ = the maximum follow-up survival rate in negative PLC group, p_1_ = the maximum follow-up survival rate in positive PLC group; PLC = pleural lavage cytology; ELCWP = European Lung Cancer Working Party; NA = not available; Pos/Neg = positive/negative; CI = confidence interval.

**Table 2:** Recurrence data of PLC in the meta-analysis.

Author/Year	Location	Patients(N)	Pos/Neg PLC	Maximum follow-up (year)	Overall recurrence (n/N)		Pleural recurrence (n/N)		Distant recurrence (n/N)	
					Pos	Neg	Pos	Neg	Pos	Neg
**Pre-resection PLC**										
Buhr /1997	Germany	342	132/210	4	96/132	35/210	22/132	4/210	78/132	23/210
Higashiyama/1997	Japan	303	41/262	5	21/41	88/262	2/41	5/262	12/41	63/262
Higashiyama/2009	Japan	679	89/590	5	56/89	NA/590	21/89	14/590	32/89	NA
Kawachi/2009	Japan	568	41/527	5	24/41	141/527	7/41	31/527	13/41	69/527
Nakamura/2009	Japan	284	13/271	5	10/13	66/271	4/13	48/271	6/13	18/271
Shintani/2009	Japan	1249	67/1182	5	32/67	384/1182	3/67	7/1182	24/67	324/1182
Taniguchi/2009	Japan	281	14/267	5	9/14	53/267	2/14	19/267	4/14	23/267
Kaneda/2012	Japan	3231	148/3083	5	NA	NA	26/148	86/3083	NA	NA
Yanagawa/2014	Japan	428	19/409	5	7/19	52/409	2/19	6/409	2/19	21/409
Mazza/2014	Italy	414	15/399	5	9/15	122/399	4/15	59/399	5/15	63/399
Nakao/2015	Japan	1572	56/1516	5	40/56	387/1516	10/56	18/1516	28/56	292/1516
Overall		9,351	635/8716		248/398	1328/5043	103/635	297/8716	204/487	896/5043
**Post-resection PLC**										
Higashiyama/1997	Japan	306	44/262	5	24/44	88/262	5/44	5/262	13/44	63/262
Taniguchi/2009	Japan	293	26/267	5	18/26	53/267	4/26	19/267	6/26	23/267
Overall		599	70/529		42/70	141/529	9/70	24/529	19/70	86/529
**Combination of pre- and post-resection PLC**										
Okumura/1991	Japan	158	23/135	3	16/23	47/135	2/23	0/135	9/23	34/135
Kameyama/2014	Japan	4171	217/3954	5	141/217	1155/3954	NA	NA	NA	NA
Overall		4329	240/4089		157/240	1202/4089	2/23	0/135	9/23	34/135

PLC = pleural lavage cytology;NA = not available; Pos = positive; Neg = negative.

### Quality assessment

The mean global quality score was 75.2%, with a range of 56.5 to 86.3% (Table 1). The design subscore had the lowest value, with only three studies that were prospective but non-randomized, and the remaining studies were retrospective. The most poorly described PLC methods included studies that did not define blinding or include a description of lavage sample conservation. The correlation was not significant between the global score and the number of patients included in the study (Spearman correlation coefficient *rs* = 0.348, *p* = 0.051). The global score was not statistically significantly different between the 20 studies performed in Japan and the 8 studies performed in other countries (mean 76.3% versus 72.4%, *p* = 0.133 by the two-sample t test).

### Meta-analysis

#### Overall survival rate of all stages

**Fig 2:**
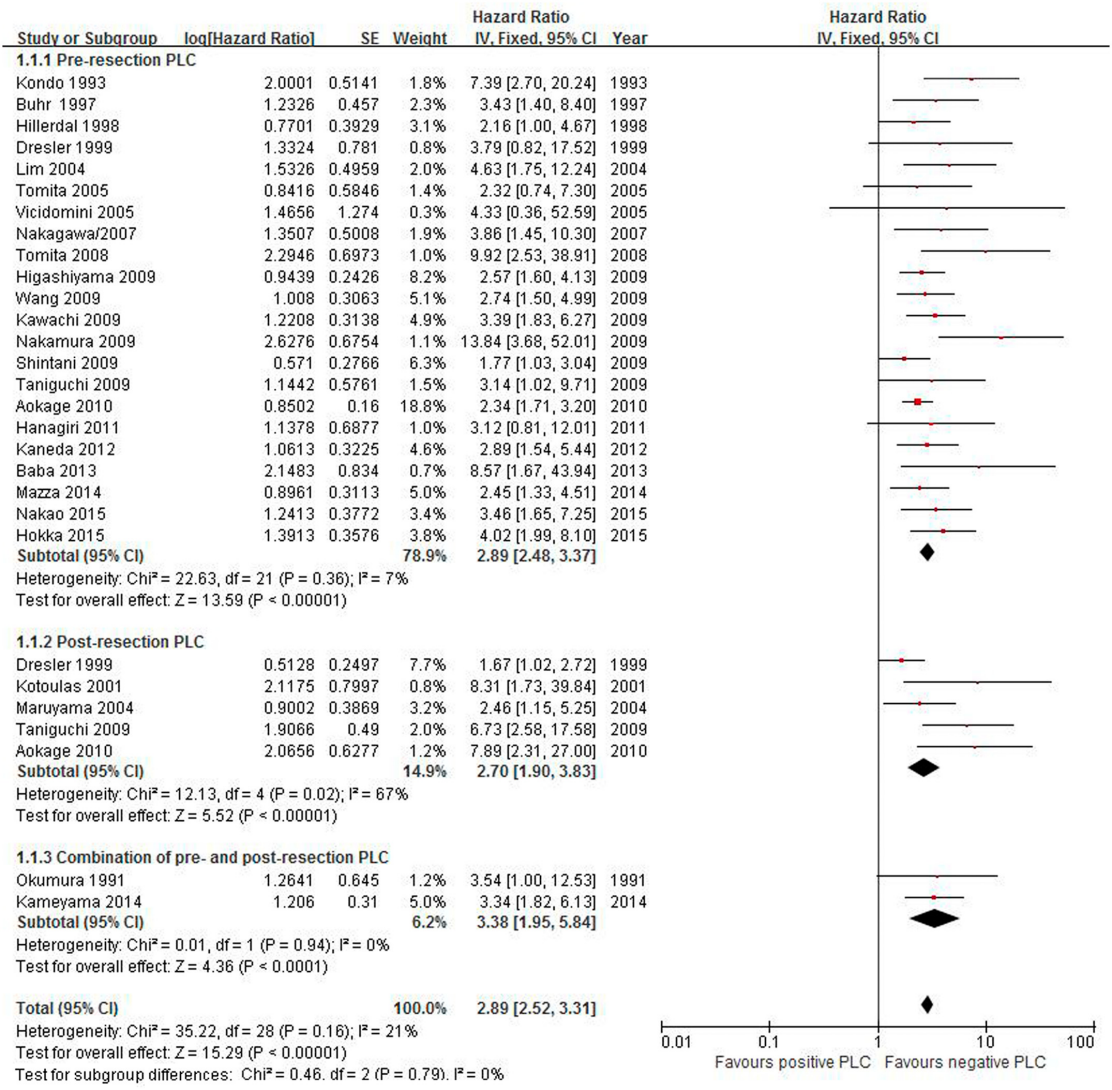
Effect of positive PLC on the overall survival rate of all stages.

Twenty-seven studies that included 19,677 patients (1,434 (7.3%) of whom were positive) reported overall survival rate of all stages. A meta-analysis demonstrated that positive pre-resection, post-resection and pooled PLC were associated with unfavorable survival: HR 2.89 (95% CI 2.48–3.37), 2.70 (1.90–3.83), and 2.89 (2.52–3.31), respectively. The test for heterogeneity was low among all studies (I^2^ = 21%, P = 0.16) (Fig 2). However, the heterogeneity in post-resection PLC was significant (I^2^ = 67%, P = 0.02). Subsequently, we performed sensitivity analyses to explore potential sources of heterogeneity. The study conducted by Dresler and colleagues [48] showed a strange CI in the forest plot, so we excluded the study and resolved the heterogeneity but did not change the results (HR 4.51, 95% CI 2.72 to 7.48; P<0.00001; P for heterogeneity = 0.21; I^2^ = 33%). We also performed a meta-analysis of the effect of positive PLC stratified by different study locations. The positive PLC patients conducted in Japan (19/27) showed a poor survival survival time (HR 3.04; 95% CI 2.69-0-3.57; P<0.00001), which similar to the experiences of remaining 8 countries (HR 2.52; 95% CI 1.94–3.28; P<0.00001).

#### Survival rate of stage I

**Fig 3:**
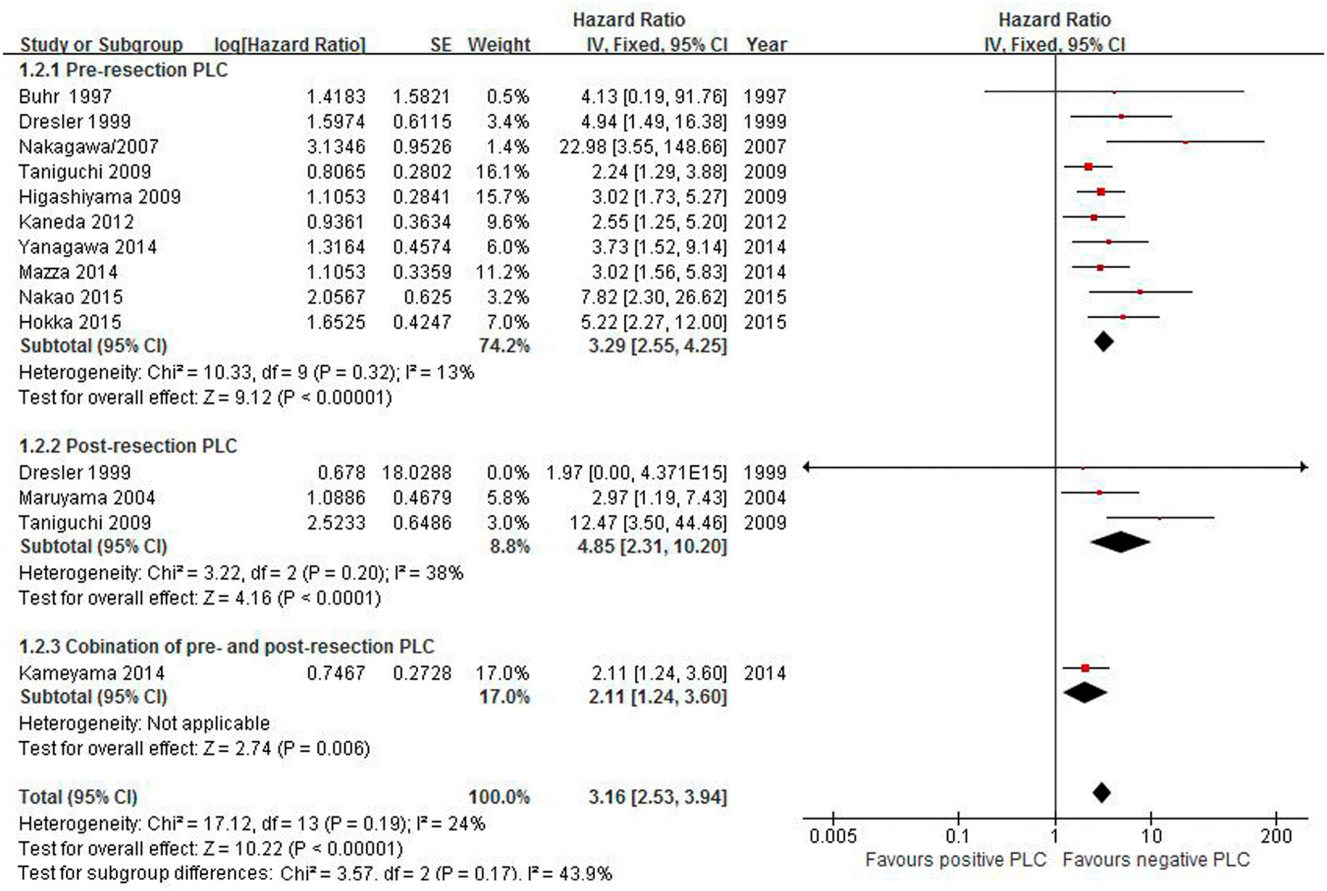
Effect of positive PLC on the survival rate of stage I lung cancer.

There were 14 studies involving 14,129 patients (869 (6.2%) of whom were positive) included in the analyses of the stage I survival rate of PLC with lung cancer resection. Positive pre-resection, post-resection and pooled PLC were associated with unfavorable survival: HR 3.29 (95% CI 2.55–4.25), 4.85 (2.31–10.20), and 3.16 (2.53–3.94), respectively. The test for heterogeneity was low among all studies (I^2^ = 24%, P = 0.19) (Fig 3).

#### Overall recurrence

**Fig 4:**
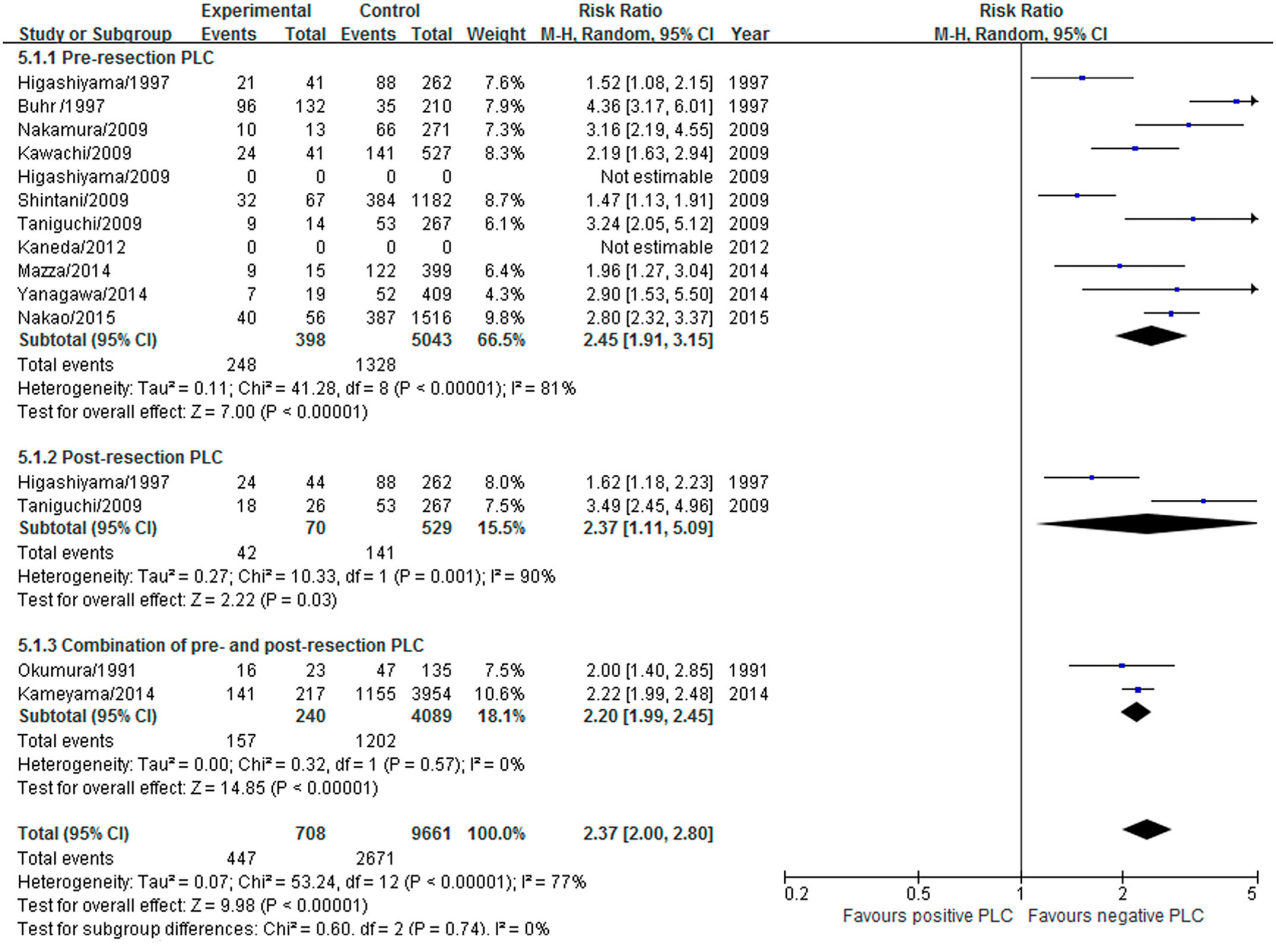
Effect of positive PLC on the overall recurrence.

Fourteen studies that included 14,279 patients reported overall recurrence data. The overall recurrence rate of positive PLC was 63.1% (447/708) and was 27.6% (2671/9661) in negative PLC. Positive pre-resection, post-resection and pooled PLC were associated with a higher likelihood of overall recurrence compared to negative PLC at maximum follow-up: RR 2.45 (95% CI 1.91–3.15), 2.37 (1.11–5.09), and 2.37 (95% CI 2.00–2.80), respectively. The test for heterogeneity was significant (I^2^ = 77%, P <0.00001) (Fig 4). In addition, the positive PLC patients conducted in Japan (12/14) showed a high recurrence (RR 2.37; 95% CI 2.03–2.78; P<0.0001), which consistent with the experiences of remaining 2 countries (RR 2.96; 95% CI 1.30–6.79; P = 0.01).

#### Pleural recurrence only

**Fig 5:**
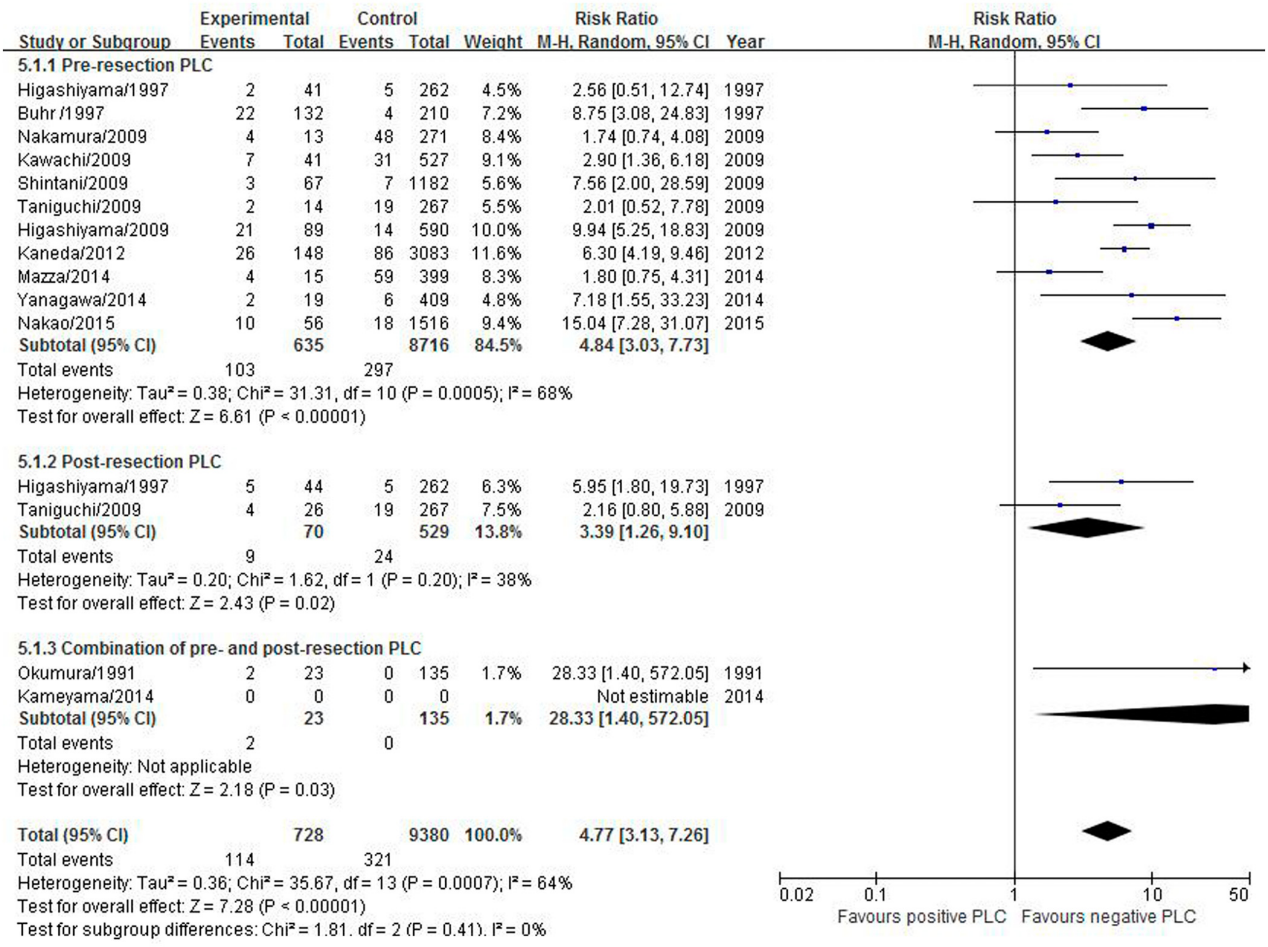
Effect of positive PLC on the pleural recurrence.

Fourteen studies including 10,108 patients reported pleural recurrence. The pooled RR was statistically significant at 4.77 (95% CI 3.13–7.26) with high heterogeneity (I^2^ = 64%, P = 0.0007) (Fig 5). Positive pre-resection PLC tended to have a higher impact on recurrence compared with positive post-resection PLC (RR 4.84 versus 3.39).

#### Distant recurrence

**Fig 6:**
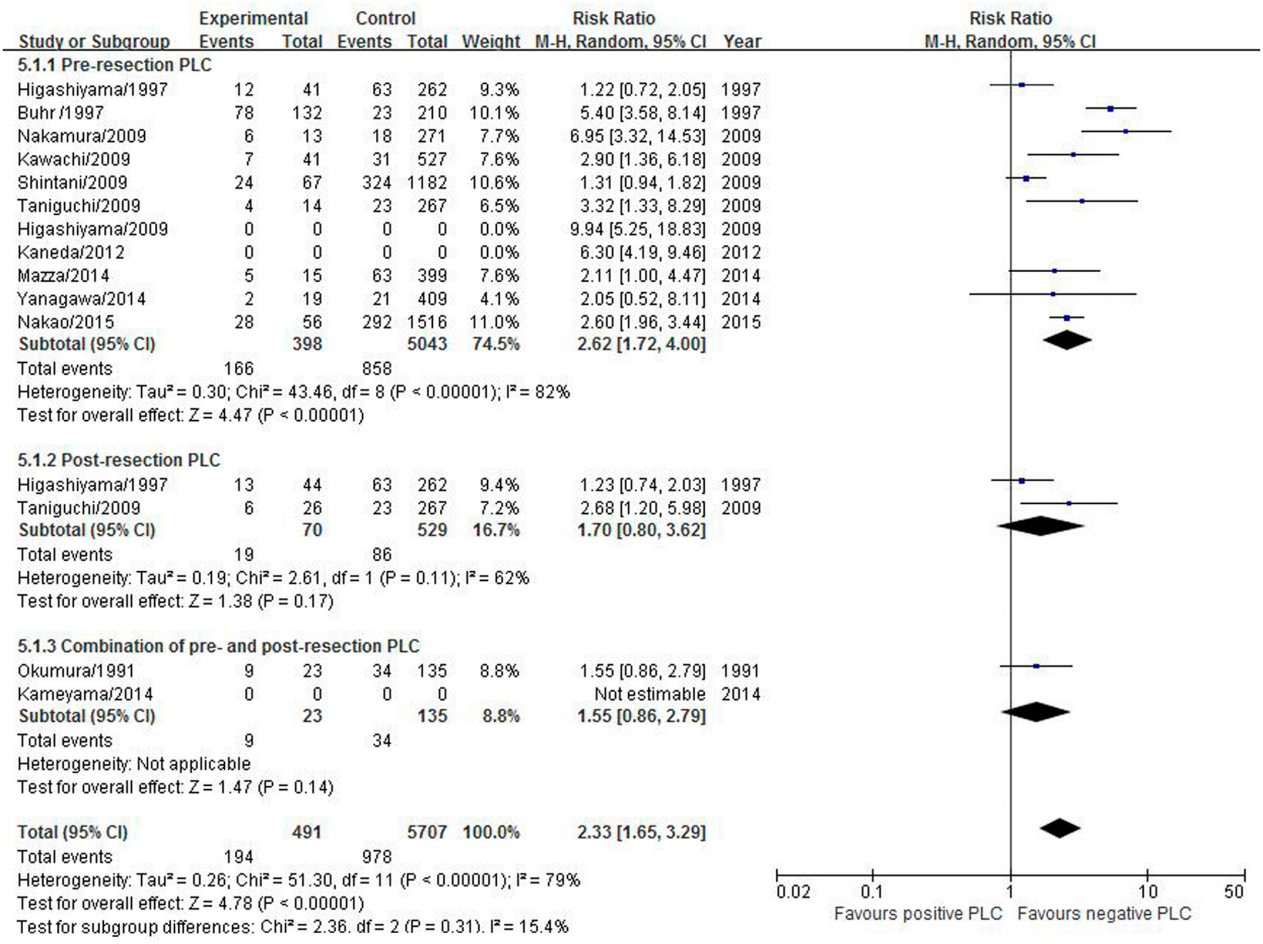
Effect of positive PLC on the distant recurrence.

Twelve studies including 6,201 patients reported distant recurrence. The pooled RR was statistically significant at 2.33 (95% CI 1.65–3.29) with high heterogeneity (I^2^ = 79%, P<0.0001). Positive pre-resection PLC was associated with an increased risk of distant recurrence (RR 2.62; 95% CI 1.72–4.00; P<0.00001), but positive post-resection PLC had no impact on distant recurrence (RR 1.70; 95% CI 0.80–3.62; P = 0.17) (Fig 6).

### Publication bias

**Fig 7:**
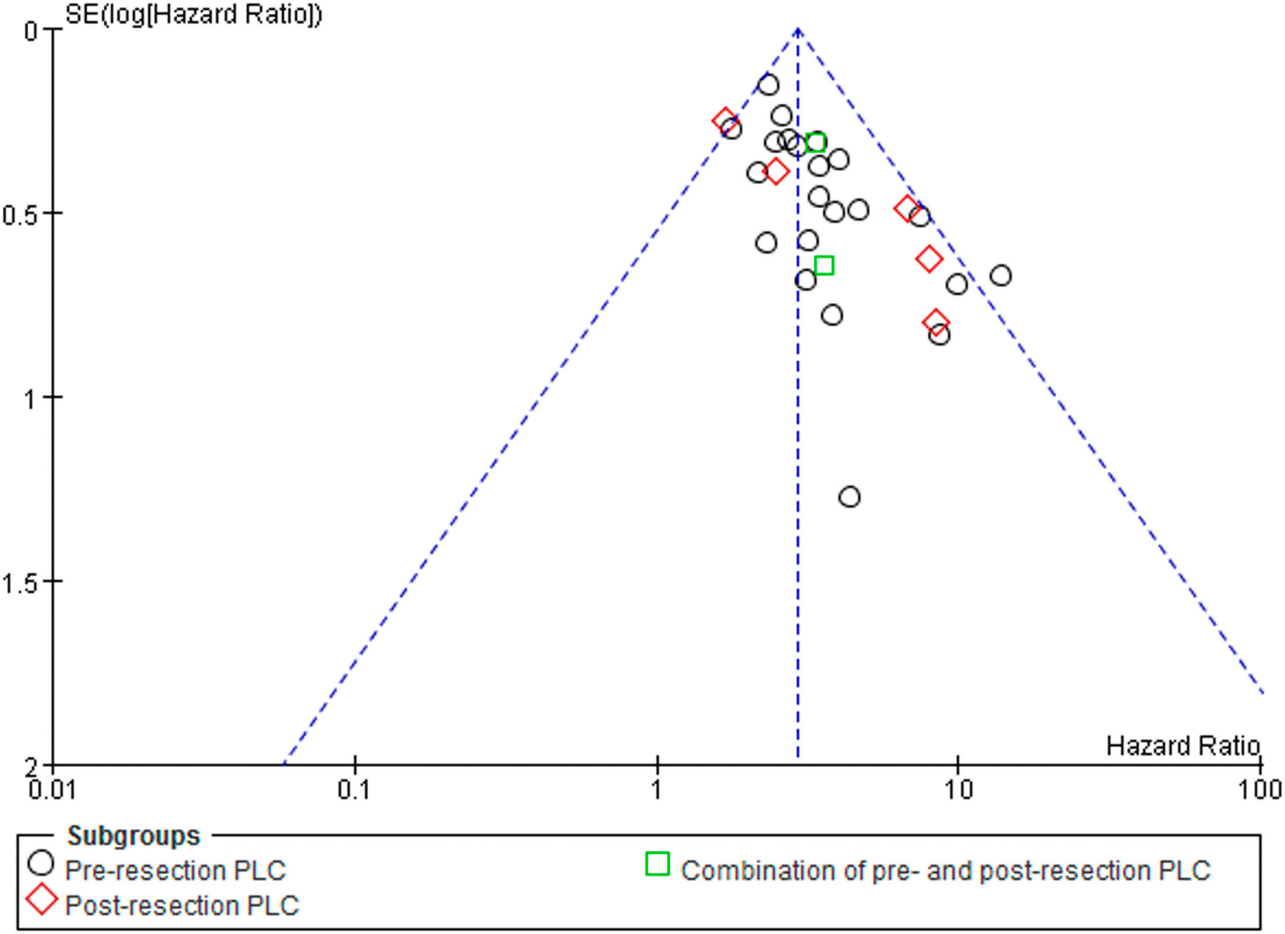
Funnel plots for publication bias for HR of overall survival rate.

Fig 7 is a funnel plot including all studies (overall survival rate) and reveals one study [56] lying outside the 95% CI. After we removed the outlier, the shapes of the funnel plots became symmetrical and the overall survival outcomes for all patients (RR 2.84; 95% CI 2.48 to 3.26) were not altered significantly.

## Discussion

NSCLC patients with positive PLC have a very poor prognosis. This meta-analysis aimed to comprehensively assess the HR of survival and the RR of recurrence for lung cancer patients having positive pre- or post-resection PLC compared to negative PLC. Our results suggest that both positive pre- and post-resection PLCs were associated not only with a higher recurrence rate but also with a poorer overall survival outcome at maximum follow-up compared with negative PLC. The HR of overall survival was 2.89 (95% CI 2.52–3.31) and the overall RR of recurrence was 2.37 (95% CI 2.00–2.80) for patients having positive PLC. In the analysis of studies that reported survival data for patients with stage I disease only, positive PLC both pre- and post-resection was also associated with unfavorable survival outcomes, and the pooled HR was 3.16 (95% CI 2.53–3.94). The recurrence and survival of positive PLC patients conducted in Japan were consistent with the experiences from other countries. The results of this meta-analysis showed that positive PLC can act as an adverse prognostic predictor for recurrence and survival in patients with lung cancer resection, regardless of whether it is performed before or after resection. However, a positive PLC result before lung resection tends to have a higher impact on survival than that of after resection, for the positive pre-resection PLC was associated with an increased risk of distant recurrence (RR 2.62; 95% CI 1.72–4.00; P<0.00001), which showed no impact on positive post-resection PLC (RR 1.70; 95% CI 0.80–3.62; P = 0.17).

The main finding of our meta-analysis was in agreement with the previously mentioned meta-analyses on this topic [6,14], which highlighted a link between unfavourable survival outcomes and positive pre-resection PLC. Our results also confirmed the findings of the meta-analysis by Saso et al,[15] which regarded pre-resection PLC were associated with not only unfavorable survival outcomes but also higher overall, local and distant recurrence. In detail, the updated meta-analysis included eight recently published articles on trials involving 11,841 patients, and some of them had large numbers of patients (>1,000). One study conducted by Kameyama et al.[63], published in 2014, involving 4,171 patients, and the authors found that 65% of patients with positive PLC and 29.2% of PLC-negative patients developed a recurrence within 5 years of surgery, which was statistically significant (P<0.0001). The 5-year survival rates were 44.5% for patients with positive PLC results and 72.8% for patients with negative PLC results (P<0.0001). In the present meta-analysis, the pooled data showed that positive pre- or post-resection PLC in patients at all stages had an unfavorable prognosis and a higher overall recurrence rate than did negative PLC. Although positive post-resection PLC was not associated with an increased risk of distant recurrence, it associated with an increased risk of higher overall and pleural recurrence compared to negative PLC. Therefore, positive pre- or post-resection PLC status was an independent factor for a poor prognosis.

The first line of treatment in patients with stage I cancer is complete lung or tumor resection, and the prognosis after resection in this category of patients is generally good. However, the survival outcome is poor if the PLC finding is positive during surgery. A previous meta-analysis [15] included nine studies showing that positive pre-resection PLC was associated with a poor survival outcome for patients with stage I cancer, and the HR was of statistical significance at 4.20. This updated meta-analysis included 14 studies (10 pre-resection and 3 post-resection). We also found that positive pre- or post-resection PLC in patients with stage I cancer had a poorer survival rate than did negative PLC, and it seems that positive post-resection PLC has a greater role in predicting survival than does positive pre-resection PLC (HR 4.85 versus 3.29). Similar results were reported by Satoh et al.[2], who found 5-year survival rates in stage I NSCLC patients of 60% in PLC-positive and 88% in PLC-negative patients. Nakagawa et al.[12] stratified their cohort of patients according to disease stage and determined that the different overall survival rate between PLC-positive and PLC-negative cases was only found in stage I NSCLC patients. The positive PLC findings seem to provide remarkable clinical information that might be used to identify a subset of stage I patients who might benefit from adjuvant treatments, including the use of local and systemic chemotherapy. Ogawa et al.[67] found that adjuvant chemotherapy after surgery tended to improve the 5-year recurrence-free survival rate by 30.9% compared with surgery alone in stage I patients with positive PLC findings. Baba et al.[62] determined that intrapleural chemotherapy with cisplatin could improve postoperative survival in PLC-positive NSCLC patients. However, these studies are small, retrospective, non-randomized, a large-scale, multi-centre, prospective randomized trial randomized-control trial is necessary.

With a mean score of 75.2%, the overall quality of all of the trials was not very good, and the main limitation of these studies is that a retrospective study design was used. On the other hand, although most studies were retrospective and only three trials were prospective, the result of the overall survival rate had low heterogeneity among the included studies. However, there was high heterogeneity within the results of recurrence, both pre-resection and post-resection, which potentially compromised the validity of our meta-analysis. The variations in inclusion criteria, patient characteristics, maximum follow-up, cytological techniques and experience of the pathologist may explain the heterogeneity.

Several potential limitations of the present meta-analysis should be taken into account. First, this meta-analysis included some clinical studies that had a small or modest sample size; for example, ten studies included no more than 200 patients each, which may cause an overestimation of the true effect compared to larger samples. Second, there was considerable heterogeneity among the studies. For example, most studies (20/28) were conducted in Japan, the number of patients varied greatly, and the maximum follow-up period and ELCWP score differed. The experience of surgeon may potentially influence the survival and recurrence outcomes, although the results from Japan were consistent with the experiences from other countries, these factors may have resulted in heterogeneity, with a potential impact on our results. Third, the inclusion of only studies published in English and Chinese may have resulted in publication bias. Furthermore, our review only included fully published studies. We did not include unpublished studies and conference abstracts because our methodology required data that was in available in full publications to perform the methodology assessment and meta-analysis.

Positive pre-resection PLC proved to be a strong independent adverse prognostic factor, positive post-resection PLC was also a strong predictor for a poor prognosis. Although 7.3% of lung cancer patients had a positive finding of PLC in our study, the presence of malignant cells in the pleural cavity without apparent pleural effusion indicates the aggressive biologic behavior of the tumor, including cell exfoliation, migration, and extravasation [60]. Therefore, we suggest that a cytological examination of PLC should be performed routinely in patients with lung cancer resection, whether before or after curative resection. A positive result may prove beneficial during the initial tumor staging process and thus subsequent management decisions. Indeed, larger tumor size, later stage, adenocarcinoma and pleural invasion were found more frequently in PLC-positive patients [7,48,64]. Since positive cytology was associated with a high pleural or distant recurrence, the adjuvant chemotherapy after surgery should focus on local (i.e. intrapleural chemotherapy) or systemic treatment according to the status of recurrence in patients with positive PLC.

In conclusion, positive pre- or post-resection PLC is associated with not only higher tumor recurrence but also unfavorable survival outcomes in all stages and particularly in stage I lung cancer patients. Positive PLC indicates subclinical dissemination of cancer cells into the pleural cavity; it means a greater possibility of recurrence or metastasis and is almost synonymous with malignant pleural effusion. Our results confirm that PLC findings are independent prognostic factors for all stages or particularly in early stage lung cancer. Further large-scale, multicenter, prospective randomized trials of ‘surgery’ versus ‘surgery and adjuvant chemotherapy’ in stage I patients with positive resection PLC may be needed.

## Supporting Information

S1 Appendix A PRISMA 2009 checklist for this meta-analysis. (DOC)

S1 Table Survival outcomes of PLC in the meta-analysis. (DOCX)

S2 Table Recurrence data of PLC in the meta-analysis. (DOCX)

## Abbreviations

PLC: pleural lavage cytology
HR: hazard ratio
CI: confidence interval
TNM: tumor nodes metastasis
NSCLC: non-small-cell lung cancer
ELCWP: European Lung Cancer Working Party
RR: risk ratio
BLC: bag lavage cytology
