# Supplementary material for: Prognostic Value of Pleural Lavage Cytology in Patients with Lung Cancer Resection: An Updated Meta-Analysis
Source: PLoS One. 2016 Jul 26;11(7):e0157518. doi: 10.1371/journal.pone.0157518 (PMC4961387; doi:10.1371/journal.pone.0157518)
Supplement: S1 Table — (DOCX) [file pone.0157518.s002.docx]

**Table 1. Survival outcomes of PLC in the meta-analysis.**

| **Author/Year** | **Location** | **Patients( N)** | **Pos/Neg**  **PLC** | **Maximum**  **follow-up (year)** | | **Overall survival rate (%)** | | | **HR** | **StageⅠsurvival rate (%)** | | **HR** | **ELCWP**  **score** |
| --- | --- | --- | --- | --- | --- | --- | --- | --- | --- | --- | --- | --- | --- |
|  |  |  |  |  |  | **Pos/Neg p-Value** | | |  | **Pos/Neg p-Value** | |  |  |
| **Pre-resection PLC** | | | | | | | | | | | | | |
| Kondo/1993 | Japan | 467 | 42/425 | | 3 | 22.9/68.7 | | <0.0001 | 7.39 | NA |  |  | 70.3 |
| Buhr /1997 | Germany | 342 | 132/210 | | 4 | 24.0/52.0 | | 0.007 | 3.43 | 35.0/69.0 | 0.037 | 4.13 | 81.3 |
| Higashiyama/1997 | Japan | 303 | 41/262 | | 5 | NA | | |  | NA |  |  | 72.8 |
| Hillerdal/1998 | Sweden | 138 | 17/121 | | 3 | 41.2/60.2 | | >0.05 | 2.16 | NA |  |  | 56.5 |
| Dresler/1999 | USA | 124 | 17/107 | | 2 | 31.0/63.0 | | 0.088 | 3.79 | 48.0/82.0 | 0.009 | 4.94 | 72.8 |
| Lim/2004 | UK | 292 | 13/279 | | 3 | 28.2/ 64.5 | | 0.002 | 4.63 | NA |  |  | 78.0 |
| Tomita/2005 | Japan | 150 | 16/134 | | 5 | 56.8 / 75.3 | | 0.15 | 2.32 | NA |  |  | 79.8 |
| Vicidomini/2005 | Italy | 84 | 19/65 | | 3 | 30.0/ 65.0 | | 0.025 | 4.33 | NA |  |  | 77.8 |
| Nakagawa/2007 | Japan | 1004 | 27/977 | | 5 | 37.0 /69.4 | | 0.007 | 3.86 | 21.8/ 86.5 | 0.001 | 22.98 | 84.5 |
| Tomita/2008 | Japan | 122 | 9/113 | | 5 | 33.3/ 83.2 | | 0.001 | 9.92 | NA |  |  | 79.8 |
| Higashiyama/2009 | Japan | 679 | 89/590 | | 5 | 43.0/66.0 | | <0.0001 | 2.57 | 57.0/ 80.0 | <0.0001 | 3.02 | 72.8 |
| Kawachi/2009 | Japan | 568 | 41/527 | | 5 | 34.4 / 64.0 | | 0.0001 | 3.39 | NA |  |  | 81.3 |
| Nakamura/2009 | Japan | 284 | 13/271 | | 5 | 12.3/ 66.0 | | <0.0001 | 13.84 | NA |  |  | 76.3 |
| Shintani/2009 | Japan | 1249 | 67/1182 | | 5 | 44.1 / 58.3 | | 0.039 | 1.77 | NA |  |  | 81.3 |
| Wang/2009 | China | 172 | 47/125 | | 5 | 26.1/ 49.2 | | <0.001 | 2.74 | NA |  |  | 67.5 |
| Taniguchi/2009 | Japan | 281 | 14/267 | | 5 | 45.0/ 72.0 | | 0.047 | 3.14 | 67.0/ 82.0 | 0.004 | 2.24 | 77.5 |
| Aokage/2010 | Japan | 2135 | 65/2070 | | 5 | CI:1.71-3.20 | | | 2.34 | NA |  |  | 80.0 |
| Hanagiri/2011 | Japan | 322 | 13/309 | | 5 | 54.7/79.0 | 0.098 | | 3.12 | NA |  |  | 72.5 |
| Kaneda/2012 | Japan | 3231 | 148/3083 | | 5 | 48.6 73.2 | <0.001 | | 2.89 | 56.8/77.0 | 0.01 | 2.55 | 80.0 |
| Baba/2013 | Japan | 386 | 17/369 | | 5 | 38.0/84.0 | <0.01 | | 8.57 | NA |  |  | 82.5 |
| Yanagawa/2014 | Japan | 428 | 19/409 | | 5 | NA |  | |  | 46.6/76.5 | <0.004 | 3.73 | 86.3 |
| Mazza/2014 | Italy | 414 | 15/399 | | 5 | 35.9/57.8 | 0.004 | | 2.45 | 42.9/69.4 | 0.001 | 3.02 | 78.8 |
| Hokka/2015 | Japan | 1317 | 46/1271 | | 5 | 28.0/61.0 | <0.0001 | | 4.02 | 39.5 /77.3 | <0.0001 | 5.22 | 75.8 |
| Nakao/2015 | Japan | 1572 | 56/1516 | | 5 | 50.6/78.0 | <0.001 | | 3.46 | 50.6 /88.9 | <0.001 | 7.82 | 72.5 |
| Overall |  | 16,064 | 983/15081 | |  |  |  | |  |  |  |  |  |
| **Post-resection PLC** | | | | | | | | | | | | | |
| Dresler/1999 | USA | 121 | 14/107 | 2 | | 43.0/58.0 | 0.04 | | 1.67 | 67.0/80.0 | 0.97 | 1.97 | 72.8 |
| Higashiyama/1997 | Japan | 306 | 44/262 | 5 | | NA |  | |  | NA |  |  | 72.8 |
| Kotoulas/2001 | Greece | 85 | 8/77 | 2 | | 52.0/90.0 | 0.0081 | | 8.31 | NA |  |  | 66.5 |
| Maruyama/2004 | Japan | 143 | 49/94 | 5 | | 55.1/ 5.1 | 0.02 | | 2.46 | 75.0/ 89.9 | 0.02 | 2.97 | 68.3 |
| Taniguchi/2009 | Japan | 293 | 26/267 | 5 | | 32.0/76.0 | <0.0001 | | 6.73 | 33.0/86.0 | <0.0001 | 12.47 | 77.5 |
| Aokage/2010 | Japan | 2140 | 70/2070 | 5 | | 22.0/ 9.0 | <0.001 | | 7.89 | NA |  |  | 80.0 |
| Overall |  | 3088 | 211/2877 |  | |  |  | |  |  |  |  |  |
| **Combination of pre- and post-resection PLC** | | | | | | | | | | | | | |
| Okumura/1991 | Japan | 158 | 23/135 | 3 | | 38.8/69.2 | <0.05 | | 3.54 | NA |  |  | 64.5 |
| Kameyama/2014 | Japan | 4171 | 217/3954 | 5 | | 44.5/72.8 | <0.0001 | | 3.34 | 61.5/77.1 | 0.0062 | 2.11 | 66.3 |
| Overall |  | 4,329 | 240/4089 |  | |  |  | |  |  |  |  |  |

HR=[p0/(1-p0)]/[p1/(1-p1)], p0= the maximum follow-up survival rate in negative PLC group，p1= the maximum follow-up survival rate in positive PLC group;

PLC= pleural lavage cytology; ELCWP= European Lung Cancer Working Party; NA = not available; Pos/Neg=positive/negative; CI=confidence interval.
