# Supplementary material for: Prognostic Value of Pleural Lavage Cytology in Patients with Lung Cancer Resection: An Updated Meta-Analysis
Source: PLoS One. 2016 Jul 26;11(7):e0157518. doi: 10.1371/journal.pone.0157518 (PMC4961387; doi:10.1371/journal.pone.0157518)
Supplement: S2 Table — (DOCX) [file pone.0157518.s003.docx]

**Table 2. Recurrence data of PLC in the meta-analysis.**

| **Author/Year** | **Location** | **Patients(N)** | **Pos/Neg**  **PLC** | **Maximum**  **follow-up**  **(year)** | **Overall recurrence**  **(n/N)** | |  | **Pleural recurrence**  **(n/N)** | |  | **Distant recurrence**  **(n/N)** | |
| --- | --- | --- | --- | --- | --- | --- | --- | --- | --- | --- | --- | --- |
|  |  |  |  |  | **Pos Neg** | |  | **Pos Neg** | |  | **Pos Neg** | |
| **Pre-resection PLC** |  |  |  |  |  |  |  |  |  |  |  |  |
| Buhr /1997 | Germany | 342 | 132/210 | 4 | 96/132 | 35/210 |  | 22/132 | 4/210 |  | 78/132 | 23/210 |
| Higashiyama/1997 | Japan | 303 | 41/262 | 5 | 21/41 | 88/262 |  | 2/41 | 5/262 |  | 12/41 | 63/262 |
| Higashiyama/2009 | Japan | 679 | 89/590 | 5 | 56/89 | NA/590 |  | 21/89 | 14/590 |  | 32/89 | NA |
| Kawachi/2009 | Japan | 568 | 41/527 | 5 | 24/41 | 141/527 |  | 7/41 | 31/527 |  | 13/41 | 69/527 |
| Nakamura/2009 | Japan | 284 | 13/271 | 5 | 10/13 | 66/271 |  | 4/13 | 48/271 |  | 6/13 | 18/271 |
| Shintani/2009 | Japan | 1249 | 67/1182 | 5 | 32/67 | 384/1182 |  | 3/67 | 7/1182 |  | 24/67 | 324/1182 |
| Taniguchi/2009 | Japan | 281 | 14/267 | 5 | 9/14 | 53/267 |  | 2/14 | 19/267 |  | 4/14 | 23/267 |
| Kaneda/2012 | Japan | 3231 | 148/3083 | 5 | NA | NA |  | 26/148 | 86/3083 |  | NA | NA |
| Yanagawa/2014 | Japan | 428 | 19/409 | 5 | 7/19 | 52/409 |  | 2/19 | 6/409 |  | 2/19 | 21/409 |
| Mazza/2014 | Italy | 414 | 15/399 | 5 | 9/15 | 122/399 |  | 4/15 | 59/399 |  | 5/15 | 63/399 |
| Nakao/2015 | Japan | 1572 | 56/1516 | 5 | 40/56 | 387/1516 |  | 10/56 | 18/1516 |  | 28/56 | 292/1516 |
| Overall |  | 9,351 | 635/8716 |  | 248/398 | 1328/5043 |  | 103/635 | 297/8716 |  | 204/487 | 896/5043 |
| **Post-resection PLC** |  |  |  |  |  |  |  |  |  |  |  |  |
| Higashiyama/1997 | Japan | 306 | 44/262 | 5 | 24/44 | 88/262 |  | 5/44 | 5/262 |  | 13/44 | 63/262 |
| Taniguchi/2009 | Japan | 293 | 26/267 | 5 | 18/26 | 53/267 |  | 4/26 | 19/267 |  | 6/26 | 23/267 |
| Overall |  | 599 | 70/529 |  | 42/70 | 141/529 |  | 9/70 | 24/529 |  | 19/70 | 86/529 |
| **Combination of pre- and post-resection PLC** | | |  |  |  |  |  |  |  |  |  |  |
| Okumura/1991 | Japan | 158 | 23/135 | 3 | 16/23 | 47/135 |  | 2/23 | 0/135 |  | 9/23 | 34/135 |
| Kameyama/2014 | Japan | 4171 | 217/3954 | 5 | 141/217 | 1155/3954 |  | NA | NA |  | NA | NA |
| Overall |  | 4329 | 240/4089 |  | 157/240 | 1202/4089 |  | 2/23 | 0/135 |  | 9/23 | 34/135 |

PLC= pleural lavage cytology;NA = not available; Pos=positive; Neg=negative.
